# 基因芯片筛选CD133^+^/CD133^-^肺腺癌细胞中新的耐药基因

**DOI:** 10.3779/j.issn.1009-3419.2014.06.01

**Published:** 2014-06-20

**Authors:** 红艳 王, 少秋 郑, 永生 涂, 雅洁 张

**Affiliations:** 1 510182 广州，广州医科大学病理教研室 Department of Pathology, Guangzhou Medical University, Guangzhou 510182, China; 2 510182 广州，广州医科大学生理教研室 Department of Physiology, Guangzhou Medical University, Guangzhou 510182, China

**Keywords:** CD133, 肺肿瘤, 耐药, 肿瘤耐药基因芯片, CD133, Lung neoplasms, Multi-drug resistant, Drug-resistant microarray

## Abstract

**背景与目的:**

肿瘤干细胞可能是肿瘤多药耐药的主要原因，CD133是目前较为公认的肿瘤干细胞标记物。本研究旨在应用功能分类基因芯片筛选CD133^+^和CD133^-^肺腺癌细胞中差异表达的肿瘤耐药基因，寻求新的肺癌耐药相关基因。

**方法:**

免疫磁珠分选法分选A549细胞，采用功能分类基因芯片筛选CD133^+^和CD133^-^肺腺癌细胞中差异表达的肿瘤耐药基因，并使用RT-qPCR验证。顺铂半数有效抑制浓度（half inhibiting concentration, IC_50_）、阿霉素IC_50_作用A549细胞48 h后，RT-qPCR检测肿瘤耐药基因*CYP2C19*、*CYP2D6*、*CYP2E1*、*GSK3α*、*PPARα*和*PPARβ*/*δ*的表达变化。

**结果:**

共筛查出31个差异表达的肿瘤耐药基因，与CD133^-^细胞相比，CD133^+^细胞有30个基因表达上调，1个基因表达下调。RT-qPCR结果与芯片一致。A549细胞经1.97 μg/mL顺铂或0.61 μg/mL阿霉素作用48 h后，*CYP2C19*、*CYP2D6*、*CYP2E1*、*GSK3α*、*PPARα*和*PPARβ*/*δ*等肿瘤耐药基因表达上调。

**结论:**

利用功能分类基因芯片筛选出31个可能与CD133^+^肺腺癌细胞耐药相关的基因，其中*CYP2C19*、*CYP2D6*、*CYP2E1*、*GSK3α*、*PPARα*和*PPARβ*/*δ*为新发现的肺癌耐药相关基因。

## Abstract (en)

**Background and objective:**

Cancer stem cells (CSCs) are responsible for multi-drug resistance in tumors. CD133 is a known biomarker of CSCs. The aim of this study is to screen for drug-resistant differentially expressed genes in CD133^+^ and CD133^-^ lung cancer cells and to identify novel lung tumor drug-resistant genes.

**Methods:**

Magnetic activated cell sorting was used to isolate CD133^+^ and CD133^-^ cells from human lung cancer cell line A549, and drug-resistant microarray was used to detect drug-resistant genes in the these cells. RT-qPCR was used to examine the expression of six lung tumor drug-resistant genes in pre-and post-chemotherapeutic A549 cells.

**Results:**

A total of 31 differentially expressed genes were screened by microarray analysis. Of these genes, 30 were upregulated and one was downregulated in CD133^+^ cells compared with CD133^-^ cells. Results were verified by RT-qPCR. *CYP2C19*, *CYP2D6*, *CYP2E1*, *GSK3α*, *PPARα*, and *PPARβ*/*δ* were significantly upregulated after the A549 cells were treated with 1.97 μg/mL DDP or 0.61 μg/mL doxorubicin for 48 h.

**Conclusion:**

The drug resistance of lung adenosarcoma may be correlated with 31 differentially expressed genes screened by drug-resistant microarray. *CYP2C19*, *CYP2D6*, *CYP2E1*, *GSK3α*, *PPARα*, and *PPARβ*/*δ* might be novel lung adenosarcoma drug-resistant genes.

肺癌是人类发病率和死亡率均居首位的恶性肿瘤。化学药物治疗是主要的治疗手段之一，但肿瘤细胞的耐药性却严重影响了抗肿瘤药物的治疗效果。肺癌细胞的耐药是多基因异常表达共同作用的结果，如P-糖蛋白（P-glycoprotein, *P-gp*）、多药耐药相关蛋白（mutidrug resistance protein, *MRP*）、乳腺癌耐药蛋白（breast cancer resistance protein, *BCRP*）和肺耐药相关蛋白（lung resistance-related protein, *LRP*）等基因的过表达，拓扑异构酶Ⅱ、谷胱甘肽-S-转移酶和蛋白激酶C的改变，另外，促进DNA修复和抑制细胞凋亡的基因表达改变以及某些癌基因的活化也可导致多药耐药^[1]^。但是有些肺癌耐药现象并不能用已知的耐药基因的表达来解释，还需要发现新的耐药相关基因。目前，国内外研究进行肿瘤耐药基因的筛选多利用生物芯片、差异显示PCR和抑制消减杂交等技术对肿瘤亲本细胞株和耐药细胞株的基因表达谱进行测定，寻找差异表达基因。肿瘤干细胞的发现使人们对肿瘤耐药的机制又有了许多新的认识，基于肿瘤干细胞理论对肺癌耐药的研究成了当今的热点。本研究以肿瘤干细胞分子标记CD133作为标志物，利用功能分类基因芯片技术对免疫磁珠分选后未经培养的CD133^+^和CD133^-^肺癌细胞的肿瘤耐药基因进行检测，筛选出差异表达基因，结合国内外文献，寻求新的肺癌耐药相关基因，为肺癌耐药的研究提供实验依据。

## 1 材料与方法

### 1.1 细胞培养

A549细胞培养在含10%新生牛血清的1640完全培养基（美国Gibco公司）中，放置在5%CO_2_、37 ℃饱和温度培养箱中培养。

### 1.2 CD133^+^和CD133^-^肺癌细胞的分离

收集对数生长期的A549细胞5×10^7^个，离心后重悬于300 μL缓冲液（2%胎牛血清、2 mmol/L EDTA、0.01% PBS），再依次加入100 μL FCR阻断剂及100 μL CD133磁性微珠（德国Miltenyi Biotec公司），充分混悬后4 ℃暗处孵育30 min，续加缓冲液洗涤、离心、重悬。将细胞悬液移入已经安装在磁性分选架的MS阳性分选柱中，待细胞悬液流出，加入2倍体积的缓冲液洗涤分选柱（洗脱下来的含CD133^-^细胞）。待洗涤缓冲液流出后加入500 μL缓冲液，装上分选柱配套的柱芯，快速推柱芯，收集流出液为CD133^+^细胞。取LD阴性分选柱安装在磁性分选架上，将MS分选过程收集的流出液加入LD分选柱中，续用缓冲液洗涤，收集流出液为CD133^-^细胞。

### 1.3 CD133^+^和CD133^-^肺癌细胞差异基因的基因芯片筛选

MS柱分选获得CD133^+^细胞及LD柱分选获得CD133^-^细胞，不经培养，直接离心沉淀细胞，每5×10^6^-10×10^6^细胞加入1 mL TRIzol试剂（Invitrogen公司）。提取的RNA用RNeasy® MinElute™纯化试剂盒（Qiagen公司）纯化。用核酸定量仪检测纯化后的RNA的浓度及纯度并进行琼脂糖凝胶电泳。cDNA合成按RT-PCR Array First Strand Kit（美国SABiosciences公司）的说明书完成。合成后的cDNA加入SuperArray功能基因芯片RT2 Profiler™ PCR Array Human Cancer Drug Resistance & Metabolism（PAHS-004A，美国SABiosciences公司）中，经荧光定量PCR仪扩增。数据分析采用ΔΔCt方法。首先计算每个处理组中的每个耐药基因的ΔCt。ΔCt=平均值Ct-管家基因平均值Ct。然后计算2个PCR Array（或两组）中每个耐药基因的ΔΔCt。ΔΔCt =ΔCt（CD133^+^细胞组）-ΔCt（CD133^-^细胞组）。最后通过2^-ΔΔCt^计算CD133^+^细胞组与CD133^-^细胞组对应基因的表达差异。筛选出2^-ΔΔCt^≥2.0或≤0.5的基因作为差异表达基因。

### 1.4 RT-qPCR验证芯片中部分差异基因表达

随机选取基因芯片中耐药差异基因*MET*、*IGF2R*、*RARG*、*PPARβ*/*δ*进行RT-qPCR分析。同时检测筛选出的新的肺癌耐药相关基因*CYP2C19*、*CYP2D6*、*CYP2E1*、*GSK3α*和*PPARα*等的表达水平。根据RT-qPCR引物设计原则，利用Primer Premier5.0进行引物设计。各差异基因和内参照GAPDH引物均由TAKARA公司合成，具体见表 1。收集分选后培养的CD133^+^细胞及CD133^-^细胞，提取RNA，方法同1.3.1。cDNA合成按TAKARA公司PrimeScript RT Reagent kit说明书完成。RT-qPCR按照Roche公司RT-qPCR试剂盒说明书，于冰上配制反应体系：FastStart Universal SYGreen Master（ROX）12.5 µL，Forward primer（10 μM）0.75 µL，Reverse primer（10 μM）0.75 µL，cDNA 1 µL，ddH_2_O 10 µL。反应条件：50 ℃ 2 min，95 ℃预变性2 min，循环参数：95 ℃ 15 s，60 ℃ 60 s，共循环40次。每个样品设2个复孔，按上述条件置RT-qPCR仪上进行扩增反应，实验重复3次。采用ΔΔCt法进行相对定量分析，结果由ABI7500软件自动生成。

**1:** RT-qPCR中各差异表达的耐药基因和内参基因*GAPHD*的引物序列 Primers for qPCR amplification

Gene	Sense strand（5'to 3'）	Antisense strand（5'to 3'）
*GAPDH*	GCACCGTCAAGGCTGAGAAC	TGGTGAAGACGCCAGTGGA
*MET*	CTCCCATCCAGTGTCTCCAGAAG	TGCAGCCCAAGCCATTCA
*IGF2R*	CCGCTAAACAGTTCGCAAGGA	CAGTTTGGGTTTCTGCCTCACA
*RARG*	CCAGCCCTACATGTTCCCAAG	CATCCTCAAACATTTCAGGGTTCTC
*PPARβ*/*δ*	CTACGGTGTTCATGCATGTGAGG	GCACTTCTGGAAGCGGCAGTA
*CYP2C19*	GGAAAACGGATTTGTGTGGGA	GGTCCTTTGGGTCAATCAGAGA
*CYP2D6*	ACCAGGCTCACATGCCCTA	TTCGATGTCACGGGATGTCAT
*CYP2E1*	ATGTCTGCCCTCGGAGTCA	CGATGATGGGAAGCGGGAAA
*GSK3α*	GGAAAGGCATCTGTCGGGG	GAGTGGCTACGACTGTGGTC
*PPARα*	ATGGTGGACACGGAAAGCC	CGATGGATTGCGAAATCTCTTGG

### 1.5 A549细胞针对顺铂（cisplatin, DDP）和阿霉素的IC_50_浓度确立

将A549细胞密度调整至5×10^4^个/mL，接种入96孔细胞培养板中，100 μL/孔，培养24 h后轻轻吸掉培养液，加入经RPMI1640培养液系列稀释的DDP或阿霉素（齐鲁制药有限公司），200 μL/孔，DDP终浓度分别为0.156 μg/mL、0.312, 5 μg/mL、0.625 μg/mL、1.25 μg/mL、2.5 μg/mL、5 μg/mL、10 μg/mL，阿霉素终浓度分别为0.062, 5 μg/mL、0.125 μg/mL、0.25 μg/mL、0.5 μg/mL、1 μg/mL、2 μg/mL、4 μg/mL，对照组不加药。各组设3个复孔，48 h后用CCK-8比色法检测细胞存活情况，测定波长为450 nm处各孔的吸光度（optical delnsity, OD）值，按以下公式计算细胞存活率：细胞存活率=（OD药物组/OD对照组）×100%。实验重复3次。通过线性拟合法计算出药物的半数有效抑制浓度（half inhibiting concentration, IC_50_）。

### 1.6 RT-qPCR检测肺癌耐药差异基因mRNA表达

收集A549细胞和DDP IC_50_和阿霉素IC_50_处理48 h后的A549细胞，抽提总RNA，cDNA合成，qPCR方法和相对定量分析同1.4。

## 2 结果

### 2.1 功能分类基因芯片结果

与CD133^-^细胞相比，CD133^+^肺腺癌细胞在84个检测的耐药基因中表达差异达两倍或以上的有31个，占36%（31/84），其中30个基因表达上调，1个基因表达下调，表达差异最为明显的基因是*RARG*与*ESR2*，它们的表达水平分别上调或下调了8.93倍和2.52倍（表 2）。这31个差异表达基因中，未被文献报道过的与肺癌耐药相关的基因有*CYP2C19*、*CYP2D6*、*CYP2E1*、*GSK3α*、*PPARα*和*PPARβ*/*δ*。

**2:** CD133^+^与CD133^-^肺腺癌细胞表达差异2倍以上的肿瘤耐药基因 Differentially expressed drug-resistant genes between CD133^+^ and CD133^-^ cells

RefSeq	Gene symbol	Gene description	Chromosomal localization	Fold change
NM_000966	*RARG*	Retinoic acid receptor, gamma	12q13	+8.93
NM_000367	*TPMT*	Thiopurine S-methyltransferase	6p22.3	+7.39
NM_019884	*GSK3α*	Glycogen synthase kinase 3 alpha	19q13.2	+5.66
NM_001800	*CDKN2D*	Cyclin-dependent kinase inhibitor 2D	19p13	+5.17
NM_017458	*MVP*	Major vault protein	16p11.2	+4.16
NM_006509	*RELB*	V-rel reticuloendotheliosis viral oncogene homolog B	19q13.32	+4.04
NM_021976	*RXRB*	Retinoid X receptor, beta	6p21.3	+3.59
NM_002503	*NFKBIB*	Nuclear factor of kappa light polypeptide gene enhancer in B-cells inhibitor, beta	19q13.1	+3.23
NM_005229	*ELK1*	ELK1, member of ETS oncogene family	Xp11.2	+3.17
NM_006238	*PPARβ*/*δ*	Peroxisome proliferator-activated receptor delta	6p21.2	+3.10
NM_000059	*BRCA2*	Breast cancer 2, early onset	13q12.3	+2.98
NM_001171	*ABCC6*	ATP-binding cassette, sub-family C (CFTR/MRP), member 6	16p13.1	+2.91
NM_000769	*CYP2C19*	Cytochrome P450, family 2, subfamily C, polypeptide 19	10q24	+2.91
NM_000122	*ERCC3*	Excision repair cross-complementing rodent repair deficiency, complementation group 3 (xeroderma pigmentosum group B complementing)	2q21	+2.81
NM_000106	*CYP2D6*	Cytochrome P450, family 2, subfamily D, polypeptide 6	22q13.1	+2.71
NM_002467	*MYC*	V-myc myelocytomatosis viral oncogene homolog (avian)	8q24.21	+2.62
NM_000876	*IGF2R*	Insulin-like growth factor 2 receptor	6q26	+2.54
NM_001437	*ESR2*	Estrogen receptor 2 (ER beta)	14q23.2	-2.52
NM_053056	*CCND1*	Cyclin D1	11q13	+2.45
NM_000546	*TP53*	Tumor protein p53	17p13.1	+2.43
NM_000038	*APC*	Adenomatous polyposis coli	5q21-q22	+2.36
NM_000392	*ABCC2*	ATP-binding cassette, sub-family C (CFTR/MRP), member 2	10q24	+2.30
NM_000321	*RB1*	Retinoblastoma 1	13q14.2	+2.27
NM_004324	*BAX*	BCL2-associated X protein	19q13.3-q13.4	+2.21
NM_005036	*PPARα*	Peroxisome proliferator-activated receptor alpha	22q13.31	+2.15
NM_004996	*ABCC1*	ATP-binding cassette, sub-family C (CFTR/MRP), member 1	16p13.1	+2.14
NM_001668	*ARNT*	Aryl hydrocarbon receptor nuclear translocator	1q21	+2.14
NM_138579	*BCL2L1*	BCL2-like 1	20q11.21	+2.13
NM_000245	*MET*	Met proto-oncogene (hepatocyte growth factor receptor)	7q31	+2.11
NM_000773	*CYP2E1*	Cytochrome P450, family 2, subfamily E, polypeptide 1	10q24.3-qter	+2.02
NM_003839	*TNFRSF11A*	Tumor necrosis factor receptor superfamily, member 11a, NFKB activator	18q22.1	+2.01
If the fold change is positive, it means up-regulation. If the fold change is negative, it means down-regulation.				

### 2.2 功能分类基因芯片中部分差异表达基因的验证

应用RT-qPCR检测了随机选取的*RARG*、*PPARβ*/*δ*、*IGF2R*、*MET*等四个差异基因和新发现的*CYP2C19*、*CYP2D6*、*CYP2E1*、*GSK3α*和*PPARα*等肺癌耐药基因在CD133^+^细胞与CD133^-^细胞中的表达，结果见图 1。与CD133^-^细胞比较，CD133^+^细胞*RARG*、*PPARβ*/*δ*、*IGF2R*、*MET*、*CYP2C19*、*CYP2D6*、*CYP2E1*、*GSK3α*、*PPARα*等基因mRNA的表达均上调。该结果与功能分类基因芯片的检测结果一致。

**1:**
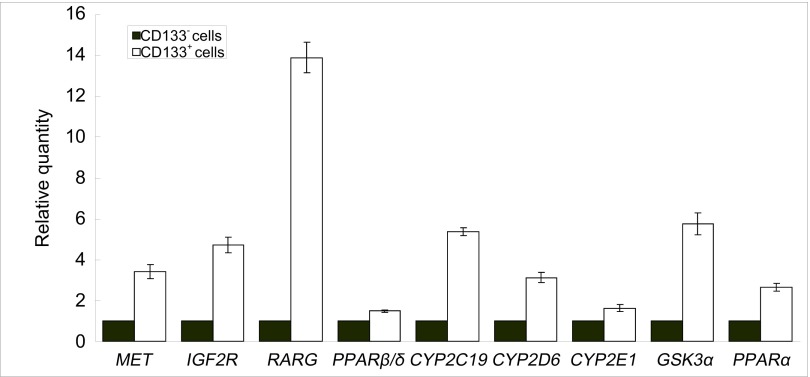
RT-qPCR验证部分差异表达基因在CD133^+^与CD133^-^细胞中的表达 Some drug-resistant genes expression between CD133^+^ and CD133^-^ cells

### 2.3 A549细胞针对DDP和阿霉素的IC_50_浓度确立

不同浓度的DDP和阿霉素对A549细胞的增殖有明显的抑制作用，通过线性拟合法计算A549细胞的DDP IC_50_为1.97 μg/mL，阿霉素IC_50_为0.61 μg/mL（表 3）。

**3:** 不同浓度DDP和阿霉素对A549细胞的抑制率（Mean±SD）（%, *n*=3） The survival rate of DDP and Doxorubicin on A549 cells (Mean±SD)(%, *n*=3)

DDP			Doxorubicin	
Concentration (μg/mL)	Survival rate of cells		Concentration (μg/mL)	Survival rate of cells
Control	100		Control	100
0.156	95.50±2.06		0.062, 5	96.40±2.52
0.312, 5	88.60±2.09		0.125	87.56±2.71
0.625	80.25±1.98		0.25	79.20±2.91
1.25	66.01±1.29		0.5	53.97±3.30
2.5	40.32±3.15		1	34.86±4.33
5	24.70±1.54		2	21.3±2.06
10	11.17±1.63		4	7.06±3.02

### 2.4 RT-qPCR对部分肺癌耐药相关基因的检测结果

A549细胞经DDP IC_50_和阿霉素IC_50_分别作用48 h后，*CYP2C19*、*CYP2D6*、*CYP2E1*、*GSK3α*、*PPARα*和*PPARβ*/*δ*等耐药相关基因表达均不同程度地上调（图 2）。

**2:**
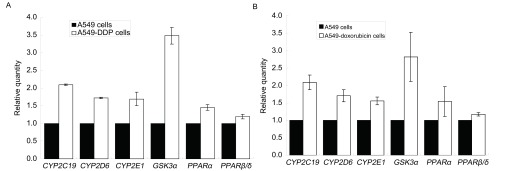
DDP IC_50_（A）和阿霉素IC_50_（B）作用前后A549细胞中肺癌耐药基因表达的变化。IC_50_：半数有效抑制浓度。 Drug-resistant genes expression levels of A549 cells in pre-and post-chemotherapy

## 3 讨论

CD133是目前使用较广泛的肿瘤干细胞标记物。研究发现，CD133^+^肺癌细胞具有干细胞样的特性，对化疗不敏感^[2-5]^。本研究以CD133作为标记物，分离CD133^+^和CD133^-^肺腺癌细胞，并筛选出二者之间的肿瘤耐药差异表达基因，为肺腺癌耐药的研究提供实验依据。

干细胞在体外培养过程中容易发生分化而失去干细胞的特性，为了维持CD133^+^细胞的未分化状态，我们将分选未经培养的CD133^+^和CD133^-^细胞直接用于后续实验。研究采用美国SuperArray公司生产的第二代功能分类基因芯片（RT2 Profiler™芯片），该芯片包含目前研究已证实与人类肿瘤耐药相关的84个基因。结果显示：在筛查的84个耐药基因中，有31个基因表达差异达两倍或两倍以上。与CD133^-^细胞相比，CD133^+^细胞有30个基因表达升高，1个基因表达降低。表达升高的基因按功能分类分别为：①药物转运蛋白相关基因，包括*ABCC1*、*ABCC2*、*ABCC6*、*MVP*；②药物代谢酶类相关基因，包括*ARNT*、*CYP2C19*、*CYP2D6*、*CYP2E1*、*GSK3α*、*TPMT*；③细胞增殖相关基因，包括*IGF2R*、*MET*、*PPARα*、*PPARβ*/*δ*、*RARG*、*RXRB*；④诱导凋亡相关基因，包括*BAX*、*BCL-XL*、*RB1*、*TP53*；⑤转录因子，包括*ELK1*、*MYC*、*NF-κBIB*、*RELB*、*TNFRSF11A*；⑥DNA修复相关基因，包括*APC*、*BRCA2*、*ERCC3*；⑦细胞周期调控基因，包括*CDKN2D*、*CCND1*。表达降低的基因是抑制细胞增殖的*ESR2*基因。这31个差异表达基因中，未被文献报道过的与肺癌耐药相关的基因有*CYP2C19*、*CYP2D6*、*CYP2E1*、*GSK3α*、*PPARα*和*PPARβ*/*δ*。

细胞色素P450（cytochrome P450, CYP450）属于血红蛋白超基因家族。作为重要的一相代谢酶，CYP450广泛参与人体内羟化、氧化、还原、水解等多种一相反应，对外源性药物、致癌化合物以及内源性物质如类固醇进行代谢。研究^[6]^表明，部分CYP450在肿瘤组织中高表达且与肿瘤的多药耐药相关。如CYP3A4/5在骨肉瘤中的高表达预示着不良的药物治疗效果^[7]^。CYP2C19、CYP2D6、CYP2E1在他莫西芬、吉非替尼、依托泊苷、长春新碱、沙利度胺、伊马替尼、环磷酰胺等多种常用抗癌药物的代谢中发挥重要作用^[8]^。现已证实CYP2C19在肝癌和结肠癌中高表达^[9]^，CYP2D6在胃癌组织中表达也较相应正常组织高^[10]^，CYP2E1在脑肿瘤、肝癌、乳腺癌、非小细胞肺癌中表达升高^[11]^，但是这些基因的表达是否与肺癌耐药相关未见报道。本研究发现CD133^+^肺腺癌细胞较CD133^-^细胞高表达CYP2C19、CYP2D6、CYP2E1，提示它们可能在CD133^+^细胞耐药过程中发挥重要作用。这些CYP450可通过增强对抗肿瘤药物的代谢作用从而减弱药物的抗肿瘤作用甚至使其灭活，使肿瘤发生耐药现象。

糖原合成酶激酶3（glycogen synthase kinase 3, GSK3）是一种多功能的丝/苏氨酸磷酸激酶，有GSK3α和GSK3β两种亚型。GSK-3的基本功能是识别和磷酸化特定序列，GSK-3α第21位点上丝氨酸的磷酸化可造成GSK-3的失活，失活的GSK-3通过调控Wnt/β-catenin、PI3K/Akt和NF-κB等多种信号传导通路参与肿瘤细胞的增殖、分化和凋亡^[12]^。Fu等^[13]^报道GSK3α mRNA及蛋白在卵巢癌耐紫杉醇细胞株中表达明显较亲本株高，认为高表达的GSK3α可能与耐药有关。Piazza等^[14]^发现在多发性骨髓瘤细胞中敲除基因*GSK3α*后，瘤细胞对bortezomib诱导的凋亡敏感性增加。本研究发现CD133^+^肺腺癌细胞较CD133^-^细胞高表达GSK3α，提示GSK3可能参与CD133^+^肺腺癌细胞的耐药。

过氧化物酶体增殖因子激活受体（peroxisome proliferator activated receptors, PPARs）是一类由配体激活的转录因子，属于核激素受体超家族成员，有PPARα、PPARβ/δ和PPARγ三种亚型。目前研究提示PPARs与肿瘤具有相关性，但PPARα和PPARβ/δ在肿瘤发生发展中的作用一直存有争议。体外实验研究发现PPARα激动剂可诱导肿瘤细胞凋亡，抑制肿瘤新生血管形成，对人子宫内膜癌细胞、卵巢癌细胞、结肠癌细胞和黑色素瘤细胞均有不同程度的抗肿瘤活性^[15]^。但在NNK诱导的鼠肺癌模型上，激活PPARα可促进肺肿瘤的发生发展^[16]^。PPARβ在肺癌组织中表达降低，PPARβ激动剂可以下调Cyclin D1和PCNA，阻滞细胞周期G_1_期从而抑制肺腺癌细胞增殖^[17]^。但也有研究^[18]^报道PPARβ/δ激活剂可以促进肺癌细胞增殖，其机制涉及通过PI3K/AKT途径上调EP4的受体PGE2，下调PTEN和增加AKT磷酸化。本研究发现CD133^+^肺腺癌细胞较CD133^-^细胞高表达PPARα和PPARβ/δ，PPARα和PPARβ/δ在肺腺癌耐药中的确切作用究竟如何？这一课题值得深入研究。

肿瘤化疗耐药是一个多基因、多环节、多途径参与的过程，可能涉及影响不同生化途径的多种遗传因子表达的改变。本研究在CD133^+^/CD133^-^肺腺癌细胞中筛选出31个可能与肺癌多药耐药相关的基因，其中*CYP2C19*、*CYP2D6*、*CYP2E1*、*GSK3α*、*PPARα*和*PPARβ*/*δ*为新发现的肺腺癌耐药相关基因，这些差异表达基因给肺腺癌耐药的研究提供了实验依据。进一步的研究将通过体内外试验证实这几个新颖的肺腺癌耐药相关基因在CD133^+^肺腺癌细胞耐药中的作用，以期为肺腺癌耐药的逆转提供新策略。
